# Human bocavirus-1 infection in hospitalized pediatric patients with acute respiratory tract infections

**DOI:** 10.1128/spectrum.02985-24

**Published:** 2025-02-25

**Authors:** Jie Tan, Ziyin Huang, Wenting Tang, Guangbing Liu, Peiqun Li, Jiaqi Wen, Lishai Mo, Chenglan Yan, Zhao Dang, Huiping Huang, Qifei Li, Chunyun Fu

**Affiliations:** 1 Department of Pediatric Respiratory Medicine, Children's Hospital, Maternal and Child Health Hospital of Guangxi Zhuang Autonomous Region, Nanning, China; 2 Medical Science Laboratory, Children's Hospital, Maternal and Child Health Hospital of Guangxi Zhuang Autonomous Region, Nanning, China; 3 Division of Neonatology, University of Miami Miller School of Medicine and Holtz Children's Hospital, Jackson Health System, Miami, Florida, USA; 4 Department of Pediatrics, University of Miami Miller School of Medicine and Holtz Children's Hospital, Jackson Health System, Miami, Florida, USA; Children's National Hospital, George Washington University, Washington, DC, USA

**Keywords:** human-bocavirus, pathogens, co-infections, pediatric patients, tNGS

## Abstract

Human bocavirus-1 (HBoV1) is an emerging viral pathogen associated with acute respiratory tract infections (ARTIs) in pediatric populations. This study aimed to evaluate the infection status and clinical characteristics of HBoV1 among hospitalized children suffering from ARTIs. A cohort of 5,021 pediatric patients with respiratory infections was analyzed using targeted next-generation sequencing to identify HBoV1 and other co-existing respiratory pathogens. Results indicated a detection rate of HBoV1 of 8.48%, predominantly among infants under 24 months, with a higher prevalence observed in males. Among the 426 children with HBoV1 detected, there were 17 cases (4%) of single HBoV1 infection, and 409 cases (96%) were co-infected with HBoV1 and other pathogens. The predominant infection pattern was HBoV1-bacteria-virus co-infection (155 cases). A total of 48 other pathogens were detected in children with HBoV1 co-infections, with rhinovirus and human herpesvirus being the most common. The median hospitalization duration for the children with HBoV1 was 8 days, and 16.26% required ICU admission for monitoring. The study offers a comprehensive analysis of the distribution of co-infecting pathogens, clinical features, and outcomes in hospitalized children with HBoV one infection.

**IMPORTANCE:**

There is currently a poor understanding of the characteristics and clinical manifestations of human bocavirus-1 (HBoV1) infection. In the past decade, few studies have thoroughly analyzed co-infecting pathogens, such as viruses, bacteria, and fungi and their patterns in relation to HBoV1. This study utilizes targeted next-generation sequencing (tNGS) technology to identify HBoV1 and other common respiratory pathogens in 5,021 hospitalized children suffering from respiratory infections in the Guangxi region. It offers a detailed analysis of the distribution of co-infecting pathogens, infection patterns, laboratory findings, clinical manifestations, imaging features, complications, and prognoses related to HBoV1 infection in this population. The study highlights the high co-infection rates associated with HBoV1 and offers important insights for diagnosing, treating, and managing children with HBoV1 infection.

## INTRODUCTION

In 2005, human bocavirus-1 (HBoV1) was first discovered in nasopharyngeal aspirate (NPA) samples from children (1). It has since been confirmed to be closely associated with respiratory infections and is now recognized as the fourth most common respiratory virus (2). HBoV consists of four genotypes: HBoV1, HBoV2, HBoV3, and HBoV4 (3). HBoV1 is primarily found in the respiratory tract, but it can also appear in the gastrointestinal tract (4). In contrast, HBoV2 to HBoV4 are mainly identified in the gastrointestinal tract (5).

HBoV1 can infect individuals of all ages, but children, particularly infants under 2 years old, are the most vulnerable (6). HBoV1 infections can occur year-round, with increased frequency in the fall and winter (7). They can arise alone or in conjunction with other pathogens, and their severity can vary from asymptomatic or mild to severe, potentially life-threatening conditions (8–11). Over the past decade, few studies have comprehensively analyzed co-infection pathogens (including viruses, bacteria, fungi, etc.) and their associated patterns in HBoV1 (12, 13). This study utilizes targeted next-generation sequencing (tNGS) technology to identify HBoV1 and other common respiratory pathogens in 5,021 hospitalized children suffering from respiratory infections in the Guangxi region. It provides a comprehensive analysis of the distribution of co-infection pathogens, infection patterns, laboratory findings, clinical manifestations, imaging features, complications, and prognoses associated with HBoV1 infection in this population.

## MATERIALS AND METHODS

### Study population

It is a retrospective analysis of 5,021 hospitalized children with acute respiratory tract infection admitted to the Maternal and Child Health Hospital of Guangxi Zhuang Autonomous Region from July 2021 to November 2023, and we collected the clinical information and testing data of the patients. Acute respiratory infection (ARI) refers to the acute clinical illness characterized by the sudden onset of respiratory symptoms caused by various pathogens (usually with a course not exceeding 21 days). The primary symptoms of ARI include coughing, sputum production, shortness of breath, a sore throat, and a runny nose. ARI mainly includes acute upper respiratory tract infections, acute bronchitis, and community-acquired pneumonia (CAP). The study was approved by the Medical Ethics Committee of Maternal and Child Health Hospital of Guangxi Zhuang Autonomous Region. Informed consent was obtained from the parents of the child patients.

### Pathogen-tNGS

The respiratory tract specimens (2,814 bronchoalveolar lavage fluids, 2,023 throat swabs, and 178 sputum samples) were collected according to the standard clinical procedure and guideline (14). Nucleic acid from the samples was extracted and purified using MagPure Pathogen DNA/RNA Kit (R6672-01B, Magen, Guangzhou, China) according to the manufacturer’s instructions. A multiplex PCR library system (Respiration100TM, KingCreate, Guangzhou, China) was used to identify 198 respiratory pathogens (80 bacteria, 79 viruses, 32 fungi, and seven other) including HBoV1. Following library preparation, the PCR products were purified using magnetic beads and subsequently amplified with primers that contained sequencing adapters and unique barcodes. The quality of the prepared library and the nucleic acid concentration were evaluated with the Qsep100 Biofragment Analyzer (Guangding Bio) and the Qubit 4.0 Fluorometer (Thermo Scientific). Finally, sequencing was conducted using the Illumina MiniSeq platform.

The raw sequencing data were subjected to quality control. Adapters and low-quality sequences were removed using the default settings of fastp v0.20.1 (15). Reads longer than 60 bp with Q30 values greater than 50% were retained to ensure high-quality data. Subsequently, the sequences were aligned to a reference database using Bowtie2 v2.4.1 (16). Reference sequences for alignment were obtained from several databases, including National Center for Biotechnology Information’s GenBank, RefSeq, and Nucleotide databases (https://www.ncbi.nlm.nih.gov). Positive signals for specific pathogens were identified using a normalized read length measured in reads per hundred thousand (RPhK). A pathogen was classified as “present” in a sample if its RPhK value was ≥10; otherwise, it was classified as “absent.” Subsequently, the clinical data of the patients were comprehensively evaluated independently by two experienced clinicians to determine the relevance of ARI and clinical potential pathogens. The evaluation included the patient’s medical history, clinical symptoms, imaging findings, tNGS results, and laboratory test results. When two doctors have a different opinion, further consult the senior doctor to reach a consensus.

### Statistical analysis

IBM SPSS Statistics 26.0 was used for statistical analyses. Quantitative data satisfying normal distribution and equal variance were expressed as mean ± standard deviation (*x* ± *s*); the independent sample *t*-test was used to compare the two groups. Median (interquartile range) was used for non-normal distribution; the non-parametric rank-sum test was used to compare groups. Qualitative data were expressed as frequency (percentages: %), and the χ test was used for comparison between groups. Differences were considered statistically significant at *P* < 0.05, and distinct differences were considered statistically significant at *P* < 0.001.

## RESULTS

### Detection rate

Among 5,021 hospitalized children with acute respiratory tract infections, tNGS detected 426 cases of HBoV1, with a detection rate of 8.48%. Among the 426 HBoV1-positive samples, there were 295 from bronchoalveolar lavage fluid, 125 from throat swabs, and six from sputum, with detection rates of 10.48% (295/2814), 6.18% (125/2023), and 3.37% (6/178), respectively.

### Pathogen detection status

**Fig 1:**
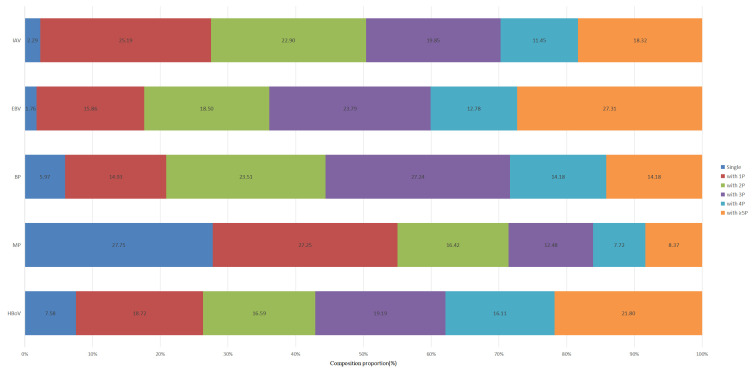
Detection of single and co-infections with various pathogens in hospitalized children with respiratory tract infections. IAV: influenza A virus; EBV: Epstein–Barr virus; BP: *Bordetella pertussis*; MP: *Mycoplasma pneumoniae*; HBoV: human bocavirus. 1P: one additional pathogen; 2P: two additional pathogens; 3P: three additional pathogens; 4P: four additional pathogens; ≥5P: five or more additional pathogens.

**Fig 2:**
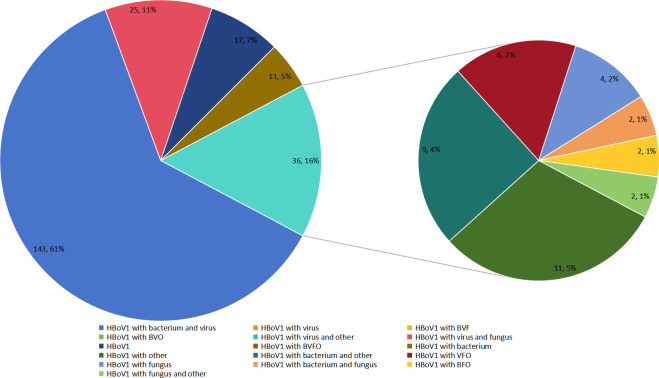
Co-infection patterns of human bocavirus. BVO: bacterium, virus, and other; VBFO: bacterium, virus, fungus, and other; BVF: bacterium, virus, and fungus; VFO: virus, fungus, and other; BFO: bacterium, fungus, and other. HBoV1: human bocavirus-1.

Among the 426 children with detected HBoV1, 17 cases (4.00%) presented with a single HBoV1 infection, while 409 cases (96.00%) exhibited co-infection with other pathogens. Specifically, there were 62 cases with one additional pathogen, 85 cases with two, 90 cases with three, 72 cases with four, and 100 cases with five or more additional pathogens. When compared to other pathogens identified by tNGS, the co-infection rate of HBoV1 is higher than that of *Mycoplasma pneumoniae* (MP), but lower than that of *Bordetella pertussis*, Epstein–Barr virus, and influenza A virus (Fig. 1). The most prevalent infection pattern was HBoV1-bacteria-virus co-infection (155/426) (Fig. 2).

### Distribution of co-infecting pathogens

**Fig 3:**
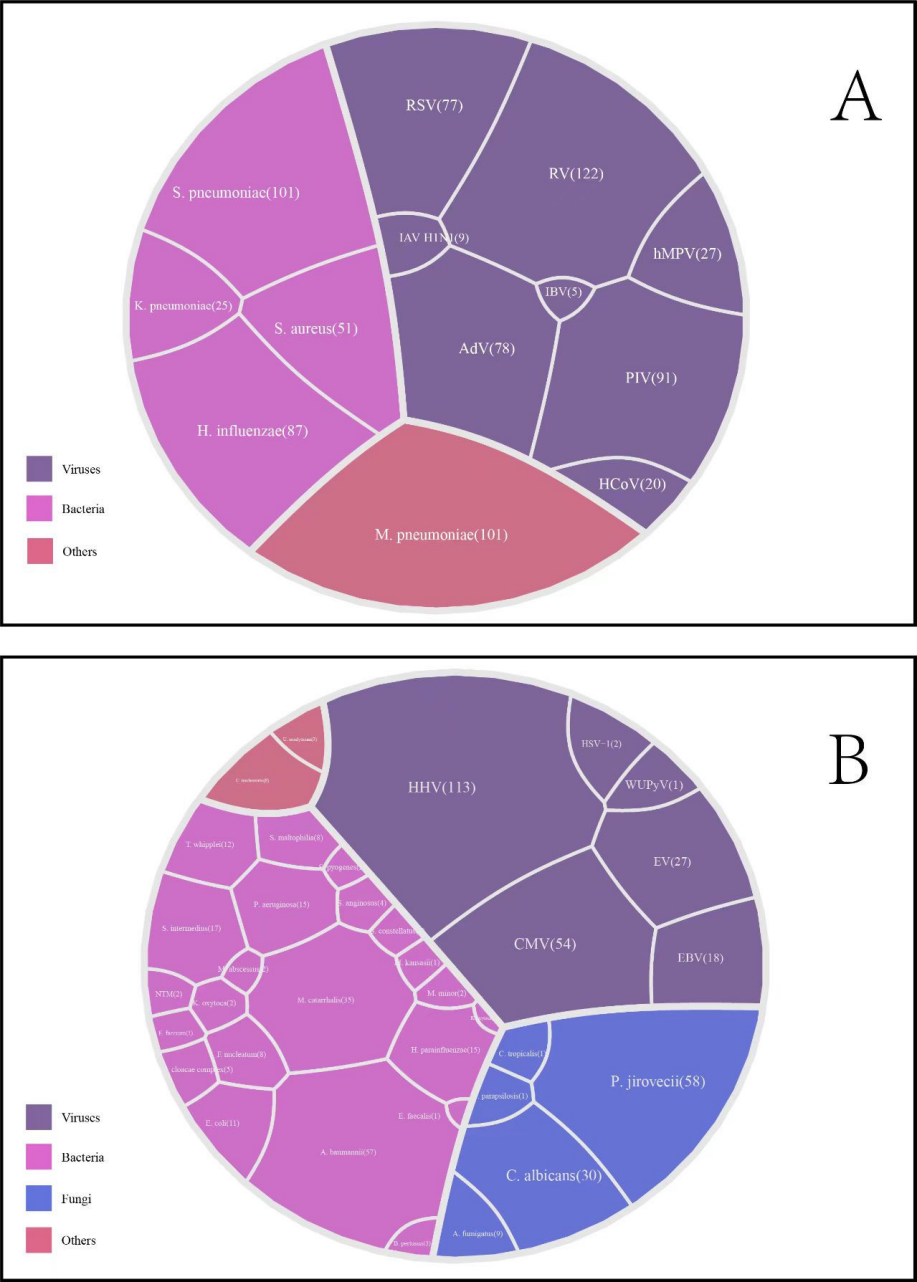
Identification and prevalence of co-infecting pathogens in children with human bocavirus infection. (A) common community-acquired pathogens; (B) opportunistic pathogens. RV: rhinovirus; PIV: parainfluenza virus; AdV: adenovirus; RSV: respiratory syncytial virus; hMPV: human metapneumovirus; HCoV: human coronavirus; IAV H1N1: influenza A virus subtype H1N1; IBV: influenza B virus; HSV-1: herpes simplex virus type 1; HHV: human herpesvirus; CMV: cytomegalovirus; EV: enterovirus; EBV: Epstein–Barr virus; WUPyV: WU polyomavirus; *S. pneumoniae: Streptococcus pneumoniae*; *H. influenzae: Haemophilus influenzae*; *S. aureus: Staphylococcus aureus*; *K. pneumoniae: Klebsiella pneumoniae*; *M. pneumoniae: Mycoplasma pneumoniae*; *A. baumannii: Acinetobacter baumannii*; *M. catarrhalis: Moraxella catarrhalis*; *S. intermedius: Streptococcus intermedius*; *H. parainfluenzae: Haemophilus parainfluenzae*; *P. aeruginosa: Pseudomonas aeruginosa*; *T. whipplei: Tropheryma whipplei*; *E. coli: Escherichia coli*; *F. nucleatum: Fusobacterium nucleatum*; *S. maltophilia: Stenotrophomonas maltophilia*; *E. cloacae complex: Enterobacter cloacae complex*; *S. anginosus: Streptococcus anginosus*; *B. pertussis: Bordetella pertussis*; *S. constellatus: Streptococcus constellatus*; *K. oxytoca: Klebsiella oxytoca*; *NTM: nontuberculous mycobacteria, S. pyogenes: Streptococcus pyogenes*; *M. abscessus: Mycobacterium abscessus*; *M. minor: Micrococcus minor*; *K. oxytoca: Klebsiella oxytoca*; *E. faecium: Enterococcus faecium*; *M. kansasii: Mycobacterium kansasii*; *E. faecalis: Enterococcus faecalis*; *P. jirovecii: Pneumocystis jirovecii*; *C. albicans: Candida albicans*; *A. fumigatus: Aspergillus fumigatus*; *C. parapsilosis: Candida parapsilosis*; *C. tropicalis: Candida tropicalis*; *C. trachomatis: Chlamydia trachomatis*; *U. urealyticum：Ureaplasma urealyticum*.

In children with mixed HBoV1 infections, a total of 48 additional pathogens were identified. This included 13 common community-acquired pathogens: eight viruses, four bacteria, and one other (Fig. 3A), as well as 35 opportunistic pathogens: six viruses, 22 bacteria, five fungi, and two others (Fig. 3B). The five most common pathogens were rhinovirus (122 cases), human herpesvirus (113 cases), *Streptococcus pneumoniae* (101 cases), MP (101 cases), and human parainfluenza virus (91 cases) (Fig. 3A). Additionally, we found enterovirus in 27 children, with a co-infection rate of 6.3% (Fig. 3B).

### Laboratory testing and clinical features

**Fig 4:**
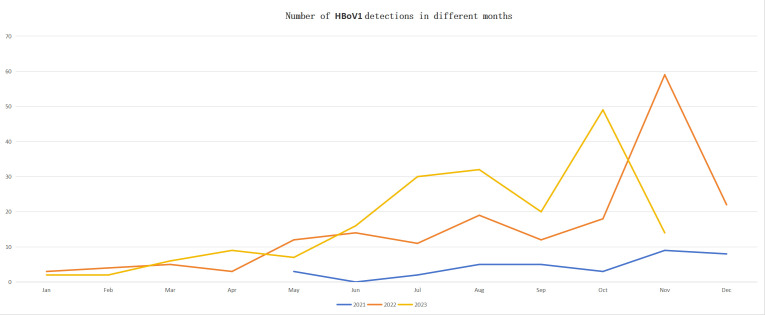
Number of HBoV1 detections in hospitalized children with respiratory tract infections across different months.

Among the 426 children infected with HBoV1, complete clinical information was collected for 406 cases. Among the 406 children, there were 271 males and 135 females, with a male-to-female ratio of 2.01:1. The age distribution ranged from 1 month to 8 years, with 122 children under 1 year, 122 children aged 1–2 years, 77 children aged 2–3 years, and 85 children over 3 years. The number of HBoV1-positive detections varied significantly across months. The second half of the year had more detections than the first half, with the peak occurring in October and November (Fig. 4).

Comparing the clinical characteristics and laboratory test results between children with single HBoV1 infection and those with mixed HBoV1 infection, as shown in Table 1, the mixed HBoV1 infection group had a higher incidence of expectoration and cough than the single HBoV1 infection group, with statistically significant differences (*P* < 0.05). Although children with a single HBoV1 infection had higher creatine kinase (CK) levels than those with co-infection, their median levels remained within the normal reference range.

**TABLE 1:** Clinical characteristics of single infection of HBoV and co-infection of HBoV^*a*^

	HBoV (*n* = 21)	Co-HBoV (*n* = 385)	*P* value
Age, year, M (IQR)	2.0 (1.3)	1.4 (1.8)	0.316
<1 year, *n* (%)	4 (19.8%)	108 (28.1%)	0.369
1–2 years, *n* (%)	5 (23.8%)	130 (33.8%)	0.346
2–3 years , *n* (%)	6 (28.6%)	57 (14.8%)	0.165
>3 years, *n* (%)	6 (28.6%)	90 (23.4%)	0.778
Sex			
male, *n* (%)	12 (57.1%)	259 (67.3%)	0.337
Clinical features, n (%)			
Fever	10 (47.6%)	255 (66.2%)	0.081
Cough	6 (28.6%)	357 (92.7%)	***<0.001
Expectoration	6 (28.6%)	333 (86.5%)	***<0.001
Gastrointestinal infection symptoms	7 (41.2%)	115 (29.6%)	0.307
Length of hospitalization, day, *M (IQR*)	6 (5)	8 (16)	0.160
Laboratory detection, M (IQR)			
WBC (×10^9^/L)	10.7 (8.2)	9.8 (5.2)	0.660
Neu (%)	59.5 (32.5)	50.2 (32.7)	0.162
Lym (%)	29.3 (34.8)	39.0 (29.8)	0.121
Eos (%)	2.1 (1.3)	1.3 (2.8)	0.105
RBC (×10^12^/L)	4.5 (0.9)	4.5 (0.7)	0.330
PLT (×10^9^/L)	363 (132)	372 (162)	0.562
Cys-C (mg/L)	0.9 (0.3)	0.9 (0.2)	0.237
CRP (mg/L)	2.6 (11.1)	4.3 (9.4)	0.281
PCT (ng/mL)	0.1 (0.14)	0.09 (0.15)	0.061
D-dimer (ng/mL FEU)	521 (622)	377 (420)	0.182
AST(U/L)	36 (10)	34 (13)	0.859
ALT(U/L)	15 (5)	17 (9)	0.720
LDH (U/L)	352 (105)	305 (79)	0.192
URE (umol/L)	25 (24)	22 (11)	0.926
CK(U/L)	125 (105)	93 (75)	*0.039
CK-MB(U/L)	29 (16)	26 (11)	0.992

^*a*^ *P<0.05, **P<0.01 and ***P<0.001 represent statistically significant differences. ALT: alanine aminotransferase; AST: aspartate aminotransferase; CK: creatine kinase; CK-MB: creatine kinase-MB; CRP: C-reactive protein; CYs-C: cystatin C; Eos: eosinophil; LDH: lactic acid dehydrogenase; LYM: lymphocyte; NEU: neutrophil; PCT: procalcitonin; PLT: platelet; RBC: red blood cell; URE: urea; WBC: white blood cell.

### Respiratory support

Among the 406 children diagnosed with HBoV1, 256 (63.05.%) required respiratory support. This support consisted of central oxygen administration in 215 children, continuous positive airway pressure in 13 children, and invasive ventilation in 28 children.

### Complications

**Fig 5:**
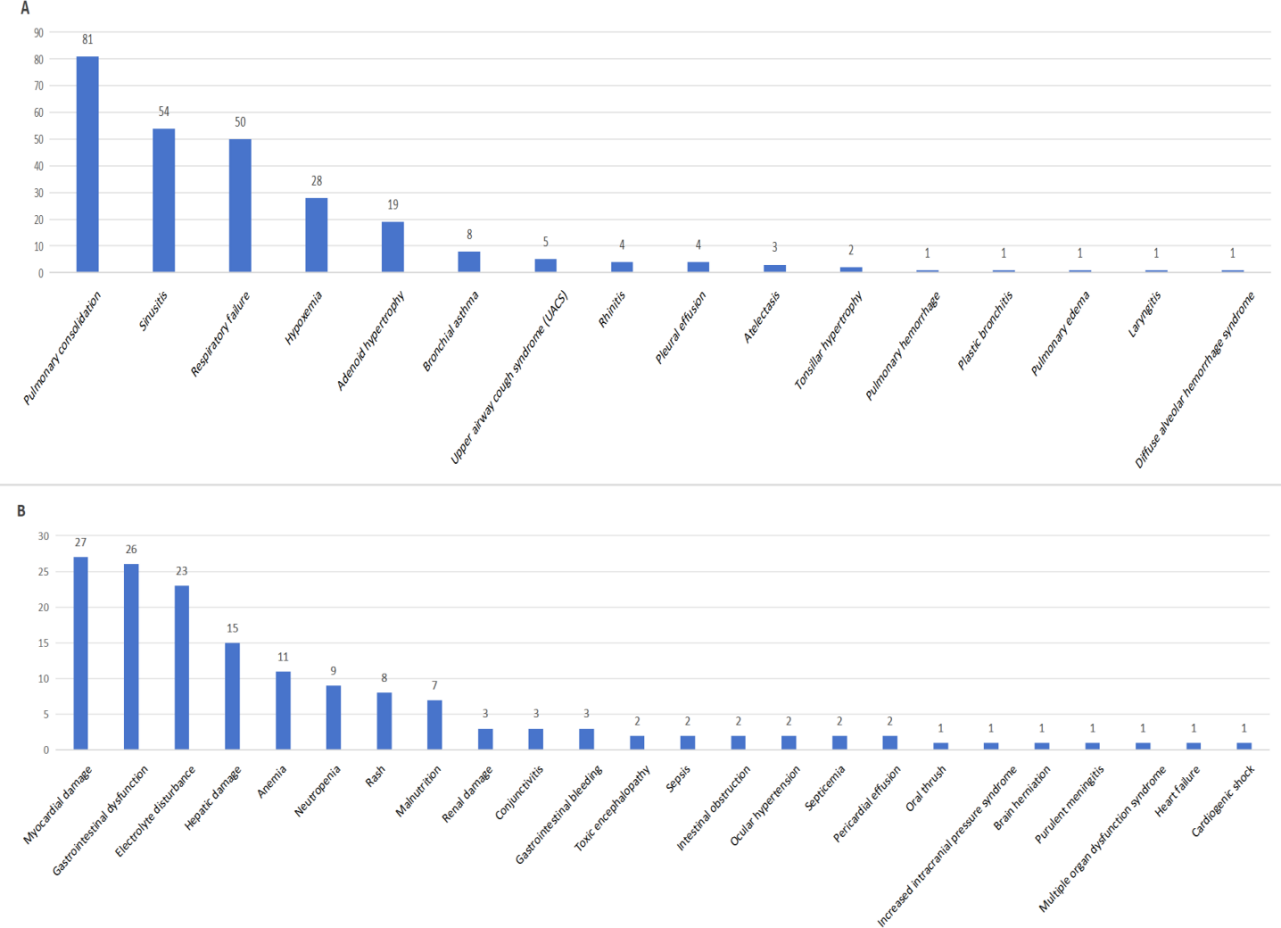
Complications of pediatric patients with human bocavirus infection. (A) Respiratory complications in patients with human bocavirus infection. (B) Other system complications in patients with human bocavirus infection.

Of the 406 children diagnosed with HBoV1, 190 (46.80%) had respiratory system complications. The most common complications included pulmonary consolidation (81 cases), sinusitis (54 cases), respiratory failure (50 cases), and hypoxemia (28 cases) (Fig. 5A). Additionally, 101 children (24.88%) had complications affecting other systems. The most common of these included myocardial damage (27 cases), gastrointestinal dysfunction (26 cases), electrolyte disorders (23 cases), and liver function damage (15 cases) (Fig. 5B).

### Prognosis

Of the 406 children infected with HBoV1, 65 (16.0%) required ICU admission for monitoring. Fifty-five of these children were admitted to the ICU due to respiratory failure, while the other 10 were admitted for serious conditions, including heart failure, shock, sepsis, toxic encephalopathy, purulent meningitis, and multi-organ dysfunction. The median hospitalization duration for the 406 children with HBoV1 was 8 days (interquartile range, 6–11 days). Following active treatment, 393 (96.80%) children recovered and were discharged, while 13 (3.20%) patients were discharged at the request of their families due to unsatisfactory treatment outcomes. No fatalities were reported.

### Comparison of clinical characteristics between ICU patients and non-ICU patients

Children in the ICU group with HBoV1 infection had a median age of 1.1 years, significantly younger than the median age of 1.6 years in the non-ICU group. Laboratory tests indicated that procalcitonin (PCT), lactic acid dehydrogenase (LDH), and CK-MB levels were significantly higher in children in the ICU than in those in the non-ICU group. Clinically, fever was more prevalent in the ICU group, which also experienced longer hospital stays (Table 2). While differences were observed between the two groups in parameters such as red blood cell (RBC) counts, lymphocyte percentages, and neutrophil percentages, their median levels remained within normal reference ranges (Table 2).

**TABLE 2:** Clinical characteristics of ICU patients and non-ICU patients^*a*^

Clinical characteristics	ICU patients (*n* = 65)	Non-ICU patients (*n* = 341)	*P* value
Age,year,M(IQR)	1.1(1.1)	1.6(2.0)	*0.028
<1 year, n(%)	19(29.3%)	93(27.3%)	0.746
1–2 years, n(%)	28(43.1%)	107(31.4%)	0.067
2–3 years, n(%)	10(15.4%)	53(15.5%)	0.974
>3 years, n(%)	8(57%)	88(25.8%)	*0.019
Sex			
male, n(%)	44(67.7%)	219(64.2%)	0.591
Clinical Features,n(%)			
Fever	53(81.5%)	212(62.2%)	*0.003
Cough	60(92.3%)	318(93.3%)	0.782
Expectoration	55(84.6%)	298(87.4%)	***<0.001
Length of hospitalization, day, *M(IQR*)	14 (10)	8(5)	***<0.001
Laboratory detection,M(IQR)			
WBC (×10^9^ /L)	9.8(5.3)	9.9(5.1)	0.592
Neu (%)	65.9 (26.3)	46.0 (31.0)	***<0.001
Lym (%)	25.3 (22.4)	40.6 (28.4)	***<0.001
Eos (%)	0.1 (1.6)	1.6 (2.8)	***<0.001
RBC (×10^12^ /L)	4.4 (0.8)	4.6 (0.7)	*0.038
PLT (×10^9^ /L)	353 (136)	376 (162)	0.278
Cys-C (mg/L)	0.9 (0.3)	0.9 (0.3)	0.246
CRP (mg/L)	6.1 (9.2)	3.8 (9.6)	0.314
PCT (ng/mL)	0.11 (0.25)	0.09 (0.14)	**0.006
D-dimer (ng/mL FEU)	452 (400)	386 (436)	0.083
AST(U/L)	36 (12)	34 (12)	0.166
ALT (U/L)	19 (11)	16 (9)	*0.011
LDH (U/L)	326(100)	305 (75)	*0.018
CK (U/L)	124 (117)	89 (68)	***<0.001
CK-MB (U/L)	29 (15)	26 (11)	*0.019

^*a*^ *P < 0.05, **P < 0.01, and ***P < 0.001 represent statistically significant differences. ALT: alanine aminotransferase; AST: aspartate aminotransferase; CYs-C: cystatin C; CK: creatine kinase; CK-MB: creatine kinase-MB; CRP: C-reactive protein; Eos: eosinophil; LDH: lactic acid dehydrogenase; LYM: lymphocyte; NEU: neutrophil; PCT: procalcitonin; PLT: platelet; RBC: red blood cell; WBC: white blood cell.

## DISCUSSION

In August 2005, Swedish researchers Allander et al. (1) first discovered HBoV in NPA samples from children. The discovery of HBoV established it as the second parvovirus known to cause disease in humans, following Parvovirus B19. HBoV is prevalent across the globe, with various genotypes targeting specific tissues, which results in different transmission routes and levels of infectivity. HBoV1 is mainly present in the respiratory tract, with a detection rate in respiratory samples ranging from approximately 1.5% to 27.0% (17–19). This study utilized tNGS to identify pathogens in 5,021 hospitalized children with respiratory infections, finding HBoV1 in 426 cases, resulting in a detection rate of 8.48%, consistent with previous research. Previous studies indicate that HBoV1 primarily affects children, particularly infants under 24 months (6), with higher detection rates observed in the fall and winter (7). This study found that among the 426 children infected with HBoV1, 122 were under 1 year old, and 122 were between 1 and 2 years old, with those under 2 years comprising 57.28% and predominantly males. Consistent with previous studies, the second half of the year showed a higher number of detections compared to the first half, especially in October and November.

HBoV1 infection can be asymptomatic or mild, but it may also cause severe and potentially life-threatening symptoms (20–22). Common symptoms of infection include cough, fever, runny nose, sputum production, wheezing, and shortness of breath (9, 21, 23, 24). Additionally, some studies have found associations between HBoV1 and the occurrence of otitis media, mumps, diarrhea, and encephalitis (25, 26). Our study found that over 80% of children infected with HBoV1 exhibited cough and expectoration, 72% had fever, 45% experienced gasping, and 24% had shortness of breath. Additionally, around 30% of the children also had gastrointestinal symptoms, mainly vomiting and diarrhea (Table 1). The study underscores the importance of considering HBoV1 as a differential diagnosis in pediatric patients presenting with respiratory and gastrointestinal symptoms. Furthermore, this study revealed that 46.80% of hospitalized children with HBoV1 experienced respiratory complications, and 24.88% faced other systemic complications. Therefore, the clinical team must be prepared for disease prevention and treatment of common and severe issues such as pulmonary consolidation, respiratory failure, myocardial damage, and liver impairment.

Relevant literature indicates that HBoV1 infection can occur alone or alongside other respiratory pathogens, especially respiratory syncytial virus, human rhinovirus, adenovirus, and parainfluenza virus, with a mixed infection rate ranging from 18% to 90% (27, 28). In this study of 426 children with HBoV1, single infections accounted for only 4%, while mixed infections comprised 96%. The rate of mixed infections is higher than previously reported (28), with HBoV1-bacteria-virus co-infection occurring frequently (36.38%, 155/426). This study shows that the five most common respiratory pathogens in cases of HBoV1 co-infection differ from those previously documented in the literature (27, 28). These differences may arise from the diversity of prevalent respiratory pathogens across various geographic regions and climates, along with the detection methods employed. Previous research shows that pathogens can interact during infections, potentially leading to complex clinical manifestations and severe disease outcomes (29, 30). Co-infection of BoV1 and enterovirus can worsen the patient’s condition, causing severe pneumonia or wheezing (31). Co-infection of HBoV1 and rhinovirus may lead to more severe respiratory symptoms and complications (32).

This study analyzed 5,021 hospitalized children with respiratory infections and found only 17 cases of single HBoV1 infection, which represents just 0.34% (17/5021). Since HBoV1 is a common respiratory virus, the low detection rate of single infections among hospitalized children raises questions about its ability to cause disease independently. In this study, we compared children with a single HBoV1 infection to those with mixed HBoV1 infections and only found that the symptoms of expectoration and cough were more common among the latter group. It is important to note that factors like birth defects, existing health issues, premature birth, and low birth weight in the children might have influenced the statistical results, which we did not exclude. In addition, the interactions between HBoV1 and other commonly detected pathogens in mixed infections, along with their effects on the body, warrant further investigation.

This study has several limitations: (i) pathogen detection relies solely on tNGS, which may not conclusively confirm the presence of a pathogen, (ii) no control group was included for comparison, and (iii) There is no evidence to support that HBoV1 is the cause of these respiratory co-infections.

In summary, the detection rate of single HBoV1 infection among hospitalized children with ARIs is merely 0.34%, and most of these children also present with co-infections involving other pathogens. This study presents a comprehensive analysis of HBoV1, focusing on its co-infection patterns, clinical manifestations, imaging features, potential complications, and overall prognosis.
